# Human-Computer Interaction: A Literature Review of Artificial Intelligence and Communication in Healthcare

**DOI:** 10.7759/cureus.73763

**Published:** 2024-11-15

**Authors:** Theo J Clay, Zephy J Da Custodia Steel, Chris Jacobs

**Affiliations:** 1 Medical Education, University of Bristol, Bristol, GBR; 2 Psychology, University of Bath, Bath, GBR

**Keywords:** artificial intelligence and education, artificial intelligence in medicine, clinical empathy, communication in healthcare, large language model (llm)

## Abstract

The integration of artificial intelligence (AI) into healthcare communication has rapidly evolved, driven by advancements in large language models (LLMs) such as Chat Generative Pre-trained Transformer (ChatGPT). This literature review explores AI's role in patient-physician interactions, particularly focusing on its capacity to enhance communication by bridging language barriers, summarizing complex medical data, and offering empathetic responses. AI's strengths lie in its ability to deliver comprehensible, concise, and medically accurate information. Studies indicate AI can outperform human physicians in certain communicative aspects, such as empathy and clarity, with models like ChatGPT and the Medical Pathways Language Model (Med-PaLM) demonstrating high effectiveness in these areas. However, significant challenges remain, including occasional inaccuracies and "hallucinations," where AI-generated content is irrelevant or medically inaccurate. These limitations highlight the need for continued refinement in AI algorithms to ensure reliability and consistency in sensitive healthcare settings. The review underscores the potential of AI as a transformative tool in health communication while advocating for further research and policy development to mitigate risks and enhance AI's integration into clinical practice.

## Introduction and background

Introduction

Since Turing’s first computer in 1944, followed by Minsky and Edmund’s first artificial intelligence (AI) model, there has been a subsequent rise in its availability and applications globally [1]. This growth became exponential through the release of the Chat Generative Pre-trained Transformer (ChatGPT) in 2018 by OpenAI, a company that researches and develops AI systems.

AI now is being used by millions of people each day, and with each input, the ability of AI grows into something that can reshape entire industries and drive innovation [2]. Once limited to producing relatively simple outputs, it has transformed into software that is capable of briskly generating highly detailed images and outputs that would have otherwise been the result of hours of manual labor. Furthermore, AI is vital for healthcare; it is capable of rapidly summarizing vast amounts of information, therefore saving valuable time.

AI is becoming increasingly essential in healthcare due to its capacity to rapidly process and summarize vast amounts of information, saving valuable time for healthcare professionals. AI assists in managing chronic conditions through predictive analytics that forecast disease progression, allowing for proactive and personalized treatment plans [2-3]. Additionally, AI-based systems can screen patients, identifying those who may not require immediate doctor intervention, which helps in optimizing healthcare resources. Recent applications of AI include aiding both doctors and patients in understanding medical conditions by providing risk assessments, supporting diagnostic imaging interpretation, and generating patient-specific insights. AI-powered algorithms are also used in remote monitoring, telemedicine, and triage systems, facilitating timely care, particularly in underserved areas [4].

Moreover, LLMs can be used to bridge language barriers with high accuracy, to help understand the concerns of the patient prior to the consultation, and to help get the most out of the consultation between the patient and the professional [5]. However, these rapid advancements come with potential drawbacks, which have been identified as inaccuracies in content generation, as well as societal and ethical implications. Similarly, studies have questioned data security and patient confidentiality, displaying how AI still lacks the human touch within communication and global healthcare usage [6]. A literature review was chosen to summarize the current research landscape of communication-focused AI use in patient and healthcare-professional interaction and to provide a comprehensive overview of the existing research. This identifies research gaps for future evaluation, and as we integrate AI into clinical work, there will be a need for research-backed policies. Clinical practice is conducted using empathy and communication skills; hence, evaluating AI is important to inform healthcare professionals of its potential use.

Research questions

This review aims to analyze the existing studies’ methodologies and demonstrate our current understanding of AI communication in healthcare. Specifically, this literature review will aim to answer the following: What are the disciplines in healthcare (within the scope of the review) in which AI technology has been studied? What is the quality of the research on AI, including a formal assessment using a valid instrument? Do AI models provide empathic communication?

## Review

Methodology

This literature review used the Medline PubMed database as the electronic database for the literature search during September 2024. Papers published between 2018 and the present look at combining the keywords "artificial intelligence", AND "health communication", OR "communication". Additionally, Google Scholar was searched for any gray literature relevant to the review. Many study types were examined within the overall longlist; however, only observational studies and experimental studies were used within the shortlist.

Studies were then excluded based on the studies’ data pool, lack of specificity to communication within the essay, reliability of the author, bias, and lack of relation to AI. Descriptive studies, articles, and posters were also removed as they did not provide enough data or insight into AI’s performance in communication. For the purpose of this review, the definition of AI is “machines or software that can perform tasks that require human intelligence and/or can mimic or simulate human cognitive processes.” Communication was defined as “the process of exchanging information, ideas, thoughts, or feelings between individuals or groups.” The authors reviewed further definitions of communication, and search terms were extended to include quality of language, comprehensibility, clarity, accuracy, and empathy.

All papers fulfilling the inclusion criteria were scored according to the Standard Quality Assessment Criteria instrument (SQACI). Validity was demonstrated for the 14-item instrument [7]. While reviewing the papers, ChatGPT-4.0/3.5 were predominantly extracted; however, additionally, Gemini 1.0 (Google AI), Med-PaLM, Fine-Tuned Language Net Pathways Language Model (Flan-PaLM), Articulate Medical Intelligence Explorer (AMIE), AI-Guide Bot, and ChatGPT-2 were also included. This was sometimes used to compare the performance of ChatGPT to other commonly used LLMs.

Results

**Figure 1:**
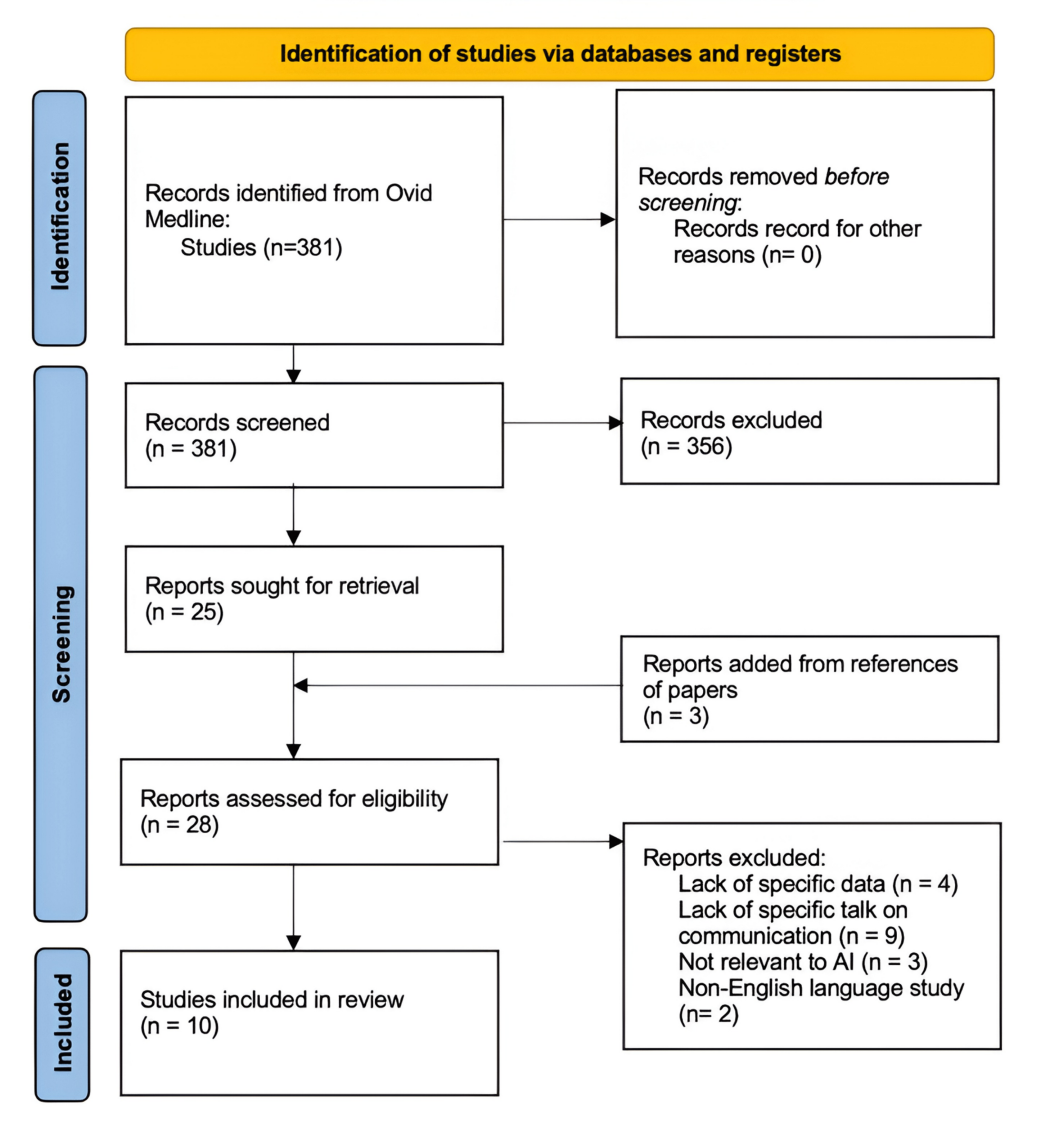
Flow diagram of the literature review process

Following the screening of 381 papers, 25 studies were longlisted. In addition to the Ovid Medline search, other studies were reviewed and selected from the reference lists in the initial 25 studies based on the excluding criteria and keywords; this was then added three to the longlist for a total of 28. Further analysis then took place to ensure papers were completely relevant to our review, which led to our short list of 10 papers. A flow chart is also provided in Figure 1, which displays our process of methodology. The 10 papers were found to have an SQACI mean score of 0.83 and a range of 0.5-1 out of 1.

Overview

All the generative AI models were able to communicate effectively with patients, generating responses that were highly comprehensible, age-appropriate, empathetic, concise, medically accurate, and of high quality. When tested against medical practitioners, AI consistently performed better in nearly all areas, highlighting its potential applications across all areas of healthcare communication, from patient-professional to interdepartmental. The quality of reviewed papers was assessed as high. However, despite this, AI was found to still have errors, sometimes producing random outputs that were incomplete, medically inaccurate, or lacked basic comprehension, as shown by [8]. This shows how, despite the automatic responses of AI within these conversations, a human touch is still needed to keep the AI in check (Table 1).

**Table 1:** Summary of results, methodology, GPT model used, and SQACI score GPT: Generative Pre-trained Model, SQACI: Standard Quality Assessment Criteria Instrument, AI: artificial intelligence, LDH: lumbar disk herniation, Flan-PaLM: Fine-Tuned Language Net Pathways Language Model, Med-PaLM: Medical Pathways Language Model, PCPs: primary care physician, OSCE: Objective Structured Clinical Examination, MRI: magnetic resonance imaging, RCT: randomized controlled trial, N/A: not applicable, SD: standard deviation, VF: very favorable, MoQL: maxim of quality, MoQN: maxim of quantity, MoR: maxim of relationship, MoM: maxim of manner

Name of paper	Author	Method	Results	AI model	Standard quality assessment criteria (3dp)
Harnessing artificial intelligence for health message generation: the folic acid message engine	Schmalzle and Wilcox (2022) [9]	A generative, pre-trained machine learning model was trained on large neutral languages. This was then fine-tuned based on tweets about folic acid using the #folicacid; it went through 2000 training steps. AI generated 1000 tweets, a random sample of 60 tweets were sampled, and a human editor curated these tweets into 30 tweets (deleting input data, false information, or problematic advice). These tweets were compared against 30 real-life tweets, and 150 adults evaluated the messages for clarity and quality and rated them 1–5, with 5 being the highest.	Clarity of AI responses: 3.58 (0.3 SD) vs. human 3.34 (0.6 SD); quality of AI responses: 3.57 (0.32 SD) vs. human 3.30 (0.53 SD).	ChatGPT-2	0.923
Large language models: are artificial intelligence-based chatbots a reliable source of patient information for spinal surgery?	Stroop et al. (2023) [10]	52 spinal surgeons, of whom 24 responded, were told to act as patients with acute LDH. They were also asked to inform an imaginary patient about the clinical picture of LDH while ignoring their background in clinical expertise. In total, 139 responses were analyzed, and the responses were then compared and analyzed.	97% were completely understandable, 93% were completely or near completely comprehensible, and 86% had satisfactory answers to the questions. Empathy: 82% were neutral, 14% were empathetic, and 3% were unempathetic or unsettling; 63% of surgeons said they improved the medical conversation.	ChatGPT	0.545
Pilot study of large language models as an age-appropriate explanatory tool for chronic pediatric conditions	Young et al. (2024) [11]	2 AI models, GPT-4 and Gemini were asked to respond to "act as a pediatrician and explain a diagnosis of a condition to an (age)-year-old in a language they can understand." The ages ranged from 5 to 17 years old, and the conditions were common chronic conditions for the children. 35 responses from each model were assessed for accuracy. Completeness, age-appropriateness, and overall quality. 3 pediatricians rated the responses from 1-5 (5 being positive).	ChatGPT-4 vs. Gemini 1.0: accuracy 4.35 and 4.55; completeness 4.25 and 4.39; age-appropriateness 3.95 and 3.26; quality 3.88 and 3.43	ChatGPT-4, Gemini 1.0	0.769
Development of AI-generated medical responses using the ChatGPT for cancer patients	Lee et al. (2024) [12]	AI-guide bot assessed by asking 100 cancer-related questions to it. Multiple medical experts then analyzed and scored comprehensibility, accuracy, and readability. 50 lay users also analyzed the responses of the AI guide bot against ChatGPT-3.5 on comprehensibility and readability using the same factors.	Medical AI-guide bot: comprehensibility (28.28/30), accuracy (34.17/40), readability (28.54/30). Non-medical AI-guidebot vs. ChatGPT: comprehensibility (28.4/30) for AI-guidebot and (26.1/30) for ChatGPT readability (28.3/30) for AI-GuideBot and (25.6/30) for ChatGPT.	ChatGPT-3.5, AI-Guidebot	0.731
Language artificial intelligence’ communicative performance quantified through the Gricean conversation theory	Nam et al. (2023) [13]	A total of 1026 dialogues were looked into; 512 involved AI sharing information, and 514 involved normal dialogue. 3365 (question-answer or initiation-correspond pairs) were classified as either a success or a failure. The failures were categorized as either failures in MoQL, MoQN, MoR, or MoM. After a chi-squared test, 71.9% of the results returned conversation success, whereas only 28.1% returned conversation failure. The proportion of conversation failure in information sharing was 25.9% compared to 30% in daily dialogue. There was a significant difference between the failure types recorded; the highest proportion was MoR (67.4%).	MoQL (be truthful): 5% failure rate; MoQN (provide as much info as required and not more): 12.8% failure rate; MoR (be relevant): 67.4% failure rate; MoM(avoid ambiguity): 14.8% failure	Unstated ChatGPT	1
Large language models encode clinical knowledge	Singhal et al. (2023) [14]	A total of 140 "long-form answer" questions were randomly selected from 3 medical question datasets, and a panel of clinicians was asked to generate expert reference answers; Flan-PaLM and Med-PaLM also generated answers. These answers were then evaluated by a different panel of clinicians and given a percentage score for appropriateness of content, bias, completeness of content, alignment with scientific consensus, as well as a non-expert lay user assessment of relevance and helpfulness.	Alignment with consensus: Flan-PaLM (61.9%), Med-PaLM (92.6%), and clinician (92.9%). Appropriateness: Flan-PaLM (83.9%), Med-PaLM (81.3%), and clinician (98.6%). Bias: Flan-PaLM (7.9%), Med-PaLM (0.8%), and clinician (1.4%). Relevance: Flan-PaLM (90.8%), Med-PaLM (94.4%), and clinician (95.9%). Helpfulness: Flan-PaLM (60.6%), Med-PaLM (80.3%), and clinician (91.1%).	Med-PaLM, Flan-PaLM	0.5
Towards conversational diagnostic AI	Tu et al.(2024) [15]	AI (AMIE) and 20 PCPs underwent OSCE examinations against 20 validated patient actors. 149 scenario packs took place under a variety of conditions. A post-questionnaire was given to the patient actors, and 23 specialist physicians reviewed and rated the consultations of both AI and PCPs. The areas assessed were across 26 categories, including management of patient concerns, explaining relevant information, information gathering, and diagnosis and management. Other aspects of communication were assessed within these categories as well.	Consultations ranked better in 24/26 of the areas with AI by actors. AI responses were rated significantly better in 28/32 of the areas by consultants. AI rated better than PCPs in clarity: 60% vs. 18% VF. Structure: 50% vs. 18% VF. Comprehensiveness: 50% vs. 15% VF. Addressing and understanding patient concerns (53% vs. 12% VF) and showing empathy (50% vs. 15% 5% VF). Building rapport: 80% vs. 50% F. Responding to emotions 30% vs. 5% VF. Using appropriate language: 98% vs. 90% F	AMIE	0.923
Patient-centered radiology reports with generative artificial intelligence: adding value to radiology reporting	Park et al (2024) [8]	AI was given prompts to summarize an MRI in a way that’s easily understandable for patients and recommend the next step. Two radiologists rated 497 lumbar and 188 cervical spine MRI consultations (685 total) from 1 to 5 (5 best). These scores were then averaged out. Two laypeople then scored the original reports vs. the AI reports and were again scored from 1 to 5 across their understanding of the content of the reports.	The average scores of the AI-generated radiologic reports were as follows: 4.86 ± 0.41 for the quality of the summary, 4.71 ± 0.60 for the compatibility of the patient-friendly reports, 4.94 ± 0.27 for the agreements of generative-AI recommendations with those made by the radiologists, and 4.84 ± 0.30 for the overall average scores across all three formats. Non-physicians results. AI 4.69 ±0.48 vs. 2.71 ± 0.73	Unstated ChatGPT	0.961
Comparing physician and artificial intelligence chatbot responses to patient questions posted to a public social media forum	Ayers et al. (2023) [16]	A cross-sectional study looked at 195 random exchanges between verified physician responses and ChatGPT responses to a question from members of the public. Evaluators looked at which response was better, the quality of information provided, and the empathy and bedside manner provided and scored from 1 to 5 (5 being the best). ChatGPT vs. physicians' responses were compared using t-tests and p<0.001. 585 evaluations took place, and the scores were then averaged.	ChatGPT responses rated better at 78.6% of the 585 evaluations and were found to be significantly better than physician responses. Mean rating: ChatGPT-4.13 vs. physicians 3.26. Proportion of responses rated good/very good quality: 78.5% for ChatGPT, 22.1% for physicians. The proportion of responses rated empathetic/very empathetic: physicians 4.6% vs. 45.1% for ChatGPT	ChatGPT-3.5, AI-Guidebot	0.958
Human–AI collaboration enables more empathic conversations in text-based peer-to-peer mental health support | nature machine intelligence	Sharma et al. (2023) [17]	RCT used. Hailey provides peer supporters with real-world feedback to those seeking help (seekers). 139 human and AI treatments vs. 161 human-only (control) treatments. Participants were asked to write supportive, empathetic responses to 10 seeker posts from a pool of 1500. The pool of 1500 was divided into 15 subsets, and the same subset was used for the control and treatment groups. 54.33% were female, 36.67% were male, 7.33% were non-binary, and 1.67% were N/A.	Human + AI rated more empathetic or equal than just human responses 62.6% of the time. Human + AI responses were preferred 62.6% of the time. Human + AI preferred 4.5% more and found a 27.01% greater empathy compared to just humans with those who experience challenges with writing responses. Human + AI increased empathy by 19.6% overall and a 38.88% increase for those with difficulty writing responses.	HAILEY	0.964

Discussion

Strengths and Limitations of the Studies

All the papers reviewed in the report had enhanced consistency, reliability, and ease of interpretation due to the use of standardized quantitative scales to rank the subjective aspects of communication. Further to this, most of the papers used large sample sizes to increase the validity of the results, with only one of the papers having less than 100 interactions analyzed [11].

The studies examined in this review were sufficiently up-to-date and relevant, owing to them all having been published since 2022, and 4 out of 10 of the papers were written in 2024 [8,11-13].

Primary research was carried out in all of the papers, allowing for unique data to be processed. In four of the papers, AI’s performance at communication compared to human controls in the form of clinicians was also assessed. This then helped to create a benchmark for which to compare the AI performance, as well as to get an insight into how the AI could be applied to health dialogue in real life.

The studies, on the other hand, did have certain limitations. While the aspects of communication were ranked on a scale, and mostly large sample sizes were used to increase the validity of results, performance in communication is subjective, and in one of the studies, only three pediatricians ranked the 35 responses from each AI model, increasing the chance of random variation within the outcomes [11]. In addition, different AIs were used in different papers. For example, one paper used ChatGPT-2, a model that is two generations behind an AI model used in another of the papers, ChatGPT-4 [9,11]. This could lead to inconsistent results as some of the data will not be about the latest AI version. One of the studies also evaluated the use of humans with AI in wellbeing communication rather than just AI alone, which could be unrepresentative of the performance of just AI [17].

Key Findings

In the important essential components, AI has certain strengths and weaknesses. It performed consistently well and even better than human controls in empathy. One research article found that 45.1% of its responses were rated empathetic/very empathetic, whereas only 4.6% of the physician responses were rated this way [16]. This is also supported by another study, in which specialist physicians rated 50% of the AMIE interactions as very favorable (VF) with regards to “showing empathy,” whereas only 15% of the primary care physician (PCP) interactions were rated VF [13]. This study also rated AMIE’s ability to build rapport as “favorable” (F) 80% of the time, whereas with PCPs, it was only F 50% of the time [15].

Finally, another paper also rated a human-supported AI well-being chatbot (HAILEY) 19.6% more empathetic than human-only trained support givers based on a validated empathy classification model developed in the paper that scored levels of empathy in responses from 0 to 6 [17,18]. Another important part of communication is clarity. ChatGPT performed very well in the study by Stroop et al., in which 97% of answers were rated “completely understandable” [10]. In fact, in a study, AMIE received a VF grade as rated by specialist physicians in 80% of the scenarios, whereas PCPs only received the VF grade in 18% of the interactions [15]. However, a different study [9] showed that there was no significant difference between the clarity of ChatGPT-2 responses and human responses -3.58 ± 0.3 vs. 3.34 ± 0.6 out of 5. This study was carried out using ChatGPT-2, so it may not have been up to date.

The AI’s ability to remain relevant, for example, "Will the AI model respond appropriately in relation to the question?" has mixed results. Med-PaLM managed to achieve similar scores of 94.4% as rated by a mixed specialist panel, whereas the clinician was able to achieve a score of 95.9% [14]. However, in Young et al.'s study, 28.1% of conversation failures occurred, with 67.4% of ChatGPT’s conversational failures being due to the maxim of relation [11]. Similarly, ChatGPT-4 scored a lower mark in the study by [11], where “age appropriateness” was evaluated. It scored 3.95/5. Another study looking at “age appropriateness” [14] found that while the clinician was able to score 91.1%, the best-performing AI Med-PaLM only got 80.3%. This said, another article rated AMIE’s “use of appropriate language” as 98% favorable compared to 90% favorable for the clinician [15].

The limitations of this study were considered to be the small timeframe of the papers that were included; however, it was judged that the newer AI models were most appropriate for review. Additionally, there were two papers with an SQACI score of 0.5, which is regarded as medium quality. No exclusion criteria based on quality score, as the research in this field can be limited to pilot studies, and this review aimed to generate a landscape of concepts around AI in communication. The mean SQACI score was 0.827, and the median score was 0.923.

Implications for the Future

A comparable literature review titled “Health Communication Meets Artificial Intelligence” similarly concludes that AI has a role in the future of patient-centered health communication [19]. It also acknowledges that there are still significant barriers to the application of these AI health interventions. Some of these barriers have been covered in more depth in this paper. While the AI’s strong performance in empathy and clarity assessments proves encouraging for AI’s application in health communication, the high conversation failure rate, predominantly down to failures in staying relevant, indicates that more work needs to be done to minimize these errors, especially due to the sensitive nature of health communication and the potential negative impact on patients. An AI without these drawbacks will be incredibly useful in the future of health communication, as it could provide 24/7 availability, more personalized interactions, and more accurate guidance to patients, lightening the workload on other healthcare staff.

## Conclusions

This literature review highlights the promising potential of AI in enhancing healthcare communication across multiple specialties, including oncology, pediatrics, radiology, primary care, and mental health. The findings suggest that AI demonstrates substantial capability in various communicative functions, signaling a prospective role for AI-driven models in healthcare settings. However, significant challenges persist, notably the issue of AI output consistency and the risk of generating irrelevant responses, or “AI hallucinations,” independent of input data. Additionally, biases inherent in the training datasets pose risks to the fairness and accuracy of AI responses. Addressing these challenges requires further research into the mechanisms through which AI processes datasets and the ability to correct inaccuracies. This, combined with a robust ethical framework, means AI communication models are poised to become an integral component of healthcare communication, potentially augmenting and complementing existing human-driven communication practices.
